# Neddylation inhibits CtIP-mediated resection and regulates DNA double strand break repair pathway choice

**DOI:** 10.1093/nar/gku1384

**Published:** 2015-01-07

**Authors:** Sonia Jimeno, María Jesús Fernández-Ávila, Andrés Cruz-García, Cristina Cepeda-García, Daniel Gómez-Cabello, Pablo Huertas

**Affiliations:** 1 Centro Andaluz de Biología Molecular y Medicina Regenerativa (CABIMER), 41092 Sevilla, Spain; 2 Departamento de Genética, Universidad de Sevilla, 41080 Sevilla, Spain

## Abstract

DNA double strand breaks are the most cytotoxic lesions that can occur on the DNA. They can be repaired by different mechanisms and optimal survival requires a tight control between them. Here we uncover protein deneddylation as a major controller of repair pathway choice. Neddylation inhibition changes the normal repair profile toward an increase on homologous recombination. Indeed, RNF111/UBE2M-mediated neddylation acts as an inhibitor of BRCA1 and CtIP-mediated DNA end resection, a key process in repair pathway choice. By controlling the length of ssDNA produced during DNA resection, protein neddylation not only affects the choice between NHEJ and homologous recombination but also controls the balance between different recombination subpathways. Thus, protein neddylation status has a great impact in the way cells respond to DNA breaks.

## INTRODUCTION

DNA is constantly challenged by physical and chemical threats that compromise its structure and function (1). Those alterations are known as DNA lesions and have to be eliminated in a process called DNA repair. Faithful restoration of the DNA molecule ensures that genomes remain stable enough during the lifetime of an organism to avoid compromising viability. To facilitate the repair of DNA, several molecular machineries have to be coordinated with the rest of the cellular metabolism. This is particularly true when repairing DNA molecules in which both strands have been broken, the so-called DNA double strand breaks (DSBs). So, upon DSB appearance a complex process known as the DNA damage response (DDR) is activated in order to sense and repair the breaks, but also to coordinate cell cycle progression, transcription, cellular metabolism, etc. (2,3). The DDR is a fast response that relies mainly in the alterations of the profiles of post-translational modifications of many different proteins, such as phosphorylation, neddylation, ubiquitylation or sumoylation (2,4).

In strictly DNA repair terms, DSBs can be repaired by a variety of pathways. Broadly, they can be divided considering the amount of homology and DNA end processing that are required during the repair process (5). Non-homologous end-joining (NHEJ) is the fast and simple religation of two DNA ends that involve no processing of the break and no homology (6). However, the ends can be processed through a mechanism known as DNA end resection, a 5′ to 3′ nucleolytic degradation of one strand of the broken DNA end (5). Such a process produces single-stranded DNA (ssDNA) 3′ overhang tails. This DNA processing can expose short (3–5 bp long) homologous sequences that can anneal facilitating the repair in a process called microhomology mediated end-joining (MMEJ; (7)). Also, DNA end resection is essential for a more complex type of repair of DSBs called homologous recombination (HR), in which long homologous sequences are used. There are different subtypes of HR repair (for review see (8)), depending if the homologous sequences are in the same molecule and in direct orientation (single strand annealing, SSA); the 3′ overhang is used to prime a replication that copies the whole chromosomal template (break-induced replication, BIR); the newly synthesized DNA is displaced from the template and reanneals to seal the break (synthesis-dependent strand annealing, SDSA); or a proper Holliday junction is formed (DSB recombination, DSBR). Thus, a broken DNA molecule can basically be repaired by six different repair mechanisms that have significantly different outcomes (1,5–8): NHEJ is fast, but the lack of a proofreading activity that ensures that the two pieces of DNA joint were originally adjacent makes it a mechanism prone to cause chromosomal rearrangements; MMEJ shares the same problems as NHEJ, plus it always causes deletions at the side of the break; SSA causes the disappearance of one of the repeats and the intervening region; BIR results in a loss of heterozygosity. SDSA and DSBR also contribute to chromosomal rearrangements when homologous sequences different to the sister chromatid are used. Thus, the regulation between all repair pathways is essential to minimize genomic instability.

The first control point for DNA repair pathway choice is the processing of the breaks. DNA end resection inhibits NHEJ and allows all the other pathways (5). So, DNA end resection is considered a primary point of DSB repair pathway choice. In eukaryotes, DNA end resection happens in two phases: a slow initial phase, catalyzed by the Mre11-Rad50-Nbs1 (MRN) complex in mammals (5), followed by a second and fast phase catalyzed by either the exonuclease Exo1 or the helicase Bloom Syndrome Protein (BLM). To initiate the process of DNA end resection, a cell cycle activation step is required in the form of the phosphorylation of the accessory protein CtIP (9,10).

Protein neddylation consists in the conjugation of the small peptide NEDD8 to protein lysines (11) in a process analog to the binding of ubiquitin or SUMO during ubiquitylation and sumoylation, respectively. Neddylation is accomplished by the subsequent action of three enzymatic activities, E1, E2 and E3 (11). First, the NEDD8 peptide is conjugated to its E1 (a dimer of UBA1 and NAE1) in an adenosine triphosphate-dependent manner to form a high-energy intermediate. Then, NEDD8 is transferred to an E2 enzyme (either UBE2M or UBE2F). The E2 will conjugate next NEDD8 to the target substrate with the help of an E3. At least three E3 enzymes, RBX1, RBX2 and RNF111, are involved in protein neddylation (12,13). Finally, the NEDD8 peptide can be removed from proteins by the activity of deneddylases. In the cell, there are two main deneddylases: a large complex known as the COP9 signalosome (CSN) and the solo acting enzyme NEDP1 (also known as SENP8) (14,15). The main substrates of protein neddylation are a family of proteins known as cullins (16), but other have been described (13,17–18). As for ubiquitylation and sumoylation, neddylation of cullins and other proteins have been involved in the DDR (13,17,19–20). Local neddylation of proteins at the vicinity of broken DNA has been shown to happen with a very fast kinetic (13,17). Moreover, DDR activation seems to be dependent on the neddylation of several histones and ubiquitin ligases RNF8 and RNF168, both essential for checkpoint activation. In addition, blocking the neddylation of Cullin 2 leads to a delayed DDR (20) and inactivation of Cullin 4 associates with a defective DNA repair (20). As for neddylation, deneddylation has also been linked with the DDR. Indeed, the COP9 signalosome (CSN) is an ATM substrate (21) and NEDP1 is responsible for elimination of RNF168 neddylation (17). Despite this relationship, little is known about the impact of protein neddylation on DNA resection and the choice between different DSB repair mechanisms.

In order to better characterize the regulatory network that controls the choice between DSB repair pathways, we took advantage of our recently published SeeSaw Reporter (SSR) that measures the balance between NHEJ and HR (22). Using a collection of small molecule inhibitors, we discovered that protein neddylation controls the choice between different repair pathways. We found that such control is dependent on the NEDD8 E2 UBE2M and E3 RNF111. RNF111/UBE2M-dependent protein neddylation has been shown to occur rapidly and locally after DNA damage (13,17). Our data show that such modification inhibits CtIP-mediated DNA end resection, suggesting that a second wave of protein deneddylation is required to activate HR. Mechanistically, we found that CtIP and its partner BRCA1 constitutively interact with neddylated proteins, and the overall neddylation status in the cell controls the interaction between them. Finally, we demonstrate that protein neddylation does not only control the choice between HR and NHEJ, but also affects the balance between different HR subpathways, specially the error-prone SSA. Thus, neddylation is a key regulatory process in the maintenance of genomic stability.

## MATERIALS AND METHODS

### Cell culture, lentiviral infection, transfection and cell survival

U2OS were grown in Dulbecco's modified Eagle's medium (Sigma-Aldrich) supplemented with 10% fetal bovine serum (Sigma-Aldrich), 2-mM L-Glutamine (Sigma-Aldrich), 100-units/ml penicillin and 100-μg/ml streptomycin (Sigma-Aldrich) supplemented with 0.5-mg/ml G418 (Gibco). Lentiviral particles were obtained as previously described (22). Cell survival assays were performed as described previously (10). Concentration of the different inhibitors used is shown in Supplementary Table S1. Information about the shRNA and siRNA used in this paper can be found in the Supplementary information. Transfection of HA-NEDD8 (Addgene) was performed using Fugene HD (Promega), following the manufacturer instructions.

### Gene conversion, SSA, NHEJ and recombination/NHEJ balance analysis

U2OS cells bearing a single copy integration of the reporters DR-GFP (Gene conversion; (23)), SA-GFP (SSA; (24)), EJ5 (NHEJ; (24)) or SSR (NHEJ/recombination balance; (22)) were used to analyze the different DSB repair pathways. In all cases, 4000 cells were plated in 96-well plates. One day after seeding, they were infected with a lentivirus harboring an I-SceI and labeled with Blue Fluorescent Protein (BFP) (25) using an M.O.I (multiplicity of infection) of 5. Six hours after infection the same volume of fresh medium was added. For the SSR screening the small molecules inhibitors were added at this point (see Supplementary information for concentrations). Cells were grown during 48 h, fixed with 4% paraformaldehyde, stained with Hoechst and washed with phosphate buffered saline (PBS) prior visualization with a fluorescent microscope for blue, green and, in the case of the SSR, red fluorescence. The repair frequency was calculated as the percentage of blue cells expressing Green Fluorescent Protein (GFP) for the DR-GFP (Gene conversion), SA-GFP (SSA), EJ2 (NHEJ) and EJ5 (MMEJ) reporters. For the HR/NHEJ balance, the ratio between green and red cells in each condition was calculated as published (22). To facilitate the comparison between experiments, this ratio was normalized with a control treated with dimethyl sulfoxide (DMSO). Those conditions that skew the balance toward an increase in NHEJ repair result in fold increase below 1. On the contrary, a net increase of this ratio (values above 1) represents an imbalance of the SSR toward HR. Data represent a minimum of three sets of duplicated experiments.

### Immunofluorescence microscopy

U2OS cells depleted for CtIP, RNF111 or UBE2M and/or were treated with MLN4924 or DMSO for 1 h as indicated in each case (see Supplementary Tables S2 and S3 for siRNAs and shRNAs used in this study). Then, cells were treated with ionizing radiation (IR; 10 Gy) or mock treated, incubated 1 h for foci formation and then collected. For Replication Protein A (RPA) foci, coverslips were treated for 5 min on ice with pre-extraction buffer (25-mM Hepes, pH 7.4, 50-mM NaCl, 1-mM ethylenediaminetetraacetic acid (EDTA), 3-mM MgCl_2_, 300-mM sucrose and 0.5% Triton X-100), then fixed with 4% paraformaldehyde (w/v) in PBS for 15 min. For RIF1 foci the treatment was carried out at room temperature, the coverslips were fixed with 4% paraformaldehyde (w/v) in PBS for 15 min, washed three times with PBS and incubated with PBS+0.25% Triton-X100 for 15 min. Then, coverslips were washed three times with PBS and blocked for at least 1 h with 5% fetal bovine serum (FBS) diluted in PBS. Cells were incubated for 8 h at 4ºC with antibodies against RPA32 or RIF1 (see Supplementary information) diluted in 5% FBS in PBS, washed twice with PBS and then incubated for 1 h at room temperature with the secondary antibody (listed in Supplementary information) diluted in 5% FBS in PBS. Coverslips were then washed twice with PBS, mounted with Vectashield mounting medium (Vector Laboratories) containing 4′,6-diamidino-2-phenylindole (DAPI) and analyzed using a Nikon NI-E microscope.

RPA foci formation was scored as the percentage of cells that have RPA foci from the total number of cells. See Supplementary Figure S1 for an example of cells positive and negative for RPA foci. The number of RIF1 foci per cell was calculated using the software Metamorph as the number of dots present in the nucleus (defined by DAPI) on those cells that show H2AX staining.

### Immunoblotting

Extracts were prepared in Laemmli buffer (4% sodium dodecyl sulphate (SDS), 20% glycerol, 120-mM Tris-HCl, pH 6.8) and proteins were resolved by SDS-polyacrylamide gel electrophoresis (PAGE) and transferred to Polyvinylidene fluoride (PVDF; Millipore) followed by immunoblotting. Western blot analysis was carried out using the antibodies listed in the Supplementary information. Results were visualized and quantified using an Odyssey Infrared Imaging System (Li-Cor).

### Immunoprecipitation

U2OS cells were harvested in lysis buffer (50-mM Tris-HCl, pH 7.4, 100-mM NaCl, 1-mM EDTA, 0.2% de Triton X-100, 1X protease inhibitors (Roche), 1X phosphatase inhibitor cocktail 1 (Sigma), NEM 3125 mg/ml (Calbiochem)). Protein extract (1 mg) was incubated at 4°C with 10 μl of anti-NEDD8 antibody (for NEDD8 immunoprecipitation) or with 10 μl of a 1:1 combination of two BRCA1 antibodies (BRCA1 immunoprecipitation; see Supplementary information for details) and with magnetic protein A Dynabeads (Novex). Beads were then washed three times with lysis buffer, and the precipitate was eluted in 25 μl of Laemmli buffer.

### Single molecule analysis of resection tracks

Single molecule analysis of resection tracks (SMART) was performed as previously described (26). U2OS cells, either treated with MNL4924 or DMSO, were grown in the presence of 10-μM bromodeoxyuridine (BrdU; GE Healthcare) for 24 h. Cultures were then irradiated (10 Gy), incubated 1 h and then harvested and embedded in low-melting agarose (Bio-Rad) followed by DNA extraction. To stretch the DNA fibers, silanized coverslips (Genomic Vision) were dipped into the DNA solution for 15 min and pulled out at a constant speed (250 μm/s). Coverslips were baked for 2 h at 60°C and incubated directly without denaturation with an anti-BrdU mouse monoclonal (Supplementary Table S4). After washing with PBS, coverslips were incubated with the secondary antibody (Supplementary Table S5). Finally, coverslips were mounted with ProLong® Gold Antifade Reagent (Molecular Probes) and stored at −20°C. DNA fibers were observed with Nikon NI-E microscope and PLAN FLOUR40×/0.75 PHL DLL objective. The images were recorded and processed with NIS ELEMENTS Nikon software. For each experiment, at least 200 DNA fibers were analyzed, and the length of DNA fibers was measured with Adobe Photoshop CS4 Extended version 11.0 (Adobe Systems Incorporated).

### Statistical analysis

Statistical significance was determined with a paired *t* student test using the PRISM software (Graphpad Software, Inc.). Statistically significant difference was labeled with one, two or three asterisks if *P* < 0.05, *P* < 0.01 or *P* < 0.001, respectively. A Mann–Whitney test was used to detect statistically significant differences between the populations of resected DNA end detected by SMART.

## RESULTS

### Screening of small inhibitors that alter the NHEJ/HR ratio

**Figure 1.**
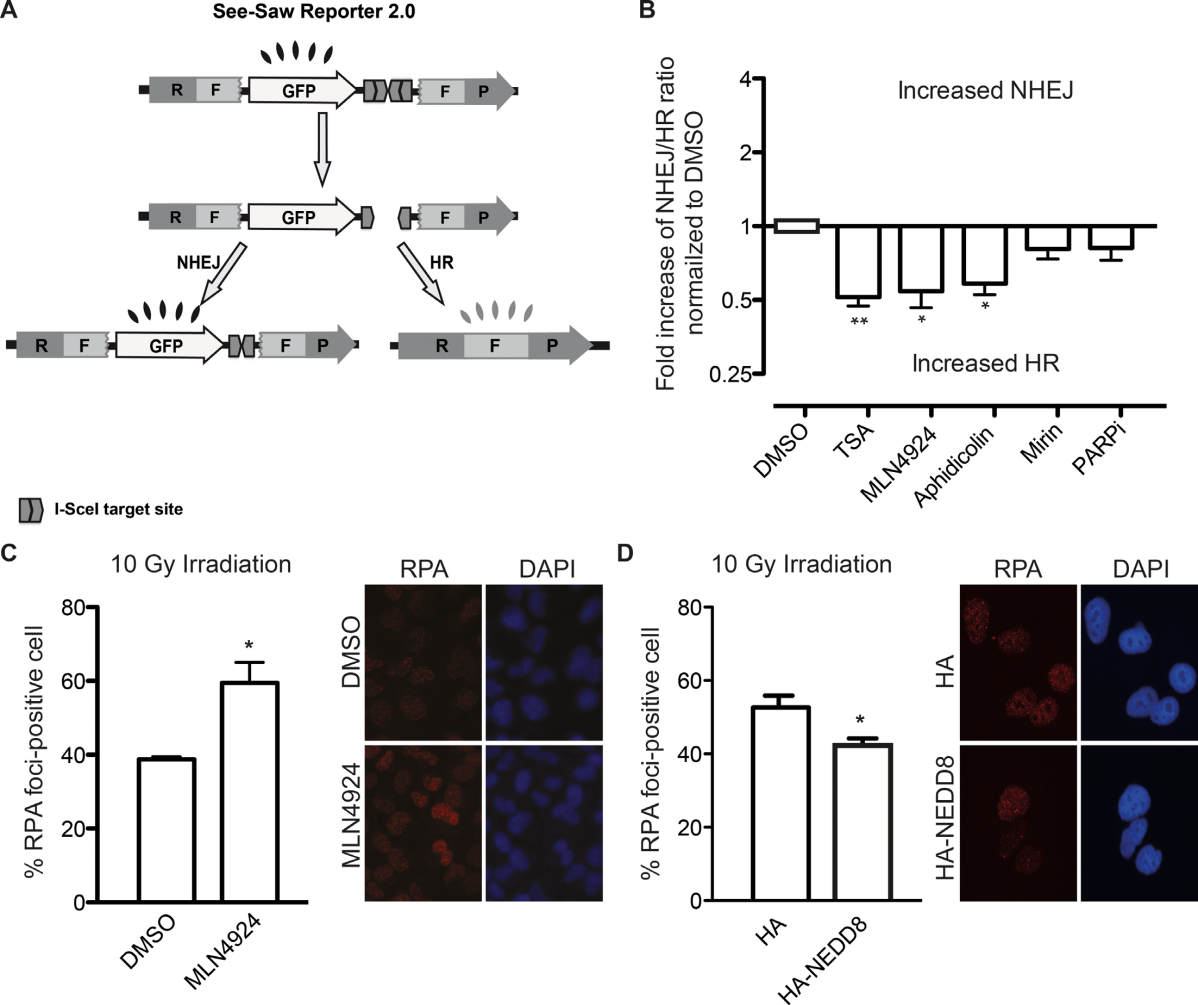
Protein neddylation inhibits CtIP-mediated DNA-end resection and HR. (**A**) Schematic representation of the SeeSaw 2.0 reporter. A GFP gene is flanked by two truncated parts of RFP gene (RF and FP) sharing 302 bp of homologous sequence. Two I-SceI-target sites were cloned at the 3′end of the GFP gene in opposite orientation. After generation of a DSB by I-SceI expression, the damage may be resolved by NHEJ, thus cells will express the GFP protein, or using the homologous sequence by HR, creating a functional RFP gene. (**B**) Effect of different inhibitors in the SSR 2.0. To measure the deviation from the balance between NHEJ and HR, the ratio between green versus red cells in each conditions was calculated. To facilitate the comparison between experiments, this ratio was normalized with control cells treated with DMSO. Those conditions that skew the balance toward an increase NHEJ result in fold increase above 1. On the contrary, a net increase of this ratio (values below 1) represents an imbalance of the SSR toward HR. Data represent a minimum of three sets of duplicated experiments. (**C**) DNA-end resection efficiency measured as the percentage of cells positive for RPA foci. Cells expressing a control shRNA (shScr) or an shRNA against CtIP (shCtIP) were pretreated with 0.2 μM of MLN4924 (MLN) or DMSO for 1 h, then irradiated (10 Gy) and incubated for an additional hour in the presence of the inhibitor. Bars represent the average and standard deviation of three independent experiments. A representative image of each case is shown. (**D**) As in (C), but cells transfected with a plasmid bearing an HA-NEDD8 gene or HA as a control. Asterisk represent statistical significance as described in the Methods section.

The balance between NHEJ and HR is essential for optimal DNA repair. We have previously designed a reporter to search for factors that alter such balance (SSR; Figure 1A; (22)). Briefly, upon creating a DSB using the nuclease I-SceI, the reporter can be repaired by either NHEJ, rendering an active GFP gene, or by a subtype of HR known as SSA, rendering an active RFP gene. So, the balance between NHEJ and HR can be calculated as the ratio between green and red cells. To look for processes that might affect the choice between DSB repair pathways, we analyzed the effect of a short list of small molecules in the SSR system (Figure 1B). As seen in the figure, we found that TSA, aphidicolin and the use of the neddylation inhibitor MLN4924 skewed the balance toward an increase in HR. Neither Mirin (a MRE11 inhibitor) nor an inhibitor of PARP rendered any effect in this reporter system. Such changes in the SSR ratio do reflect a complex reality in terms of DSB repair pathway choice. In fact, such observations might be explained either by an increase in SSA, a reduction in NHEJ or a combination of both phenomena. It can even reflect a net increase in both pathways but more accentuated for SSA or a decrease in both mechanisms but stronger in NHEJ.

The unbalance toward HR upon neddylation inhibition was surprising. Neddylation, as sumoylation and ubiquitylation, has been reported at the sites of breaks (4,13,17,19). However, contrary to the increase in HR observed by neddylation inhibition (Figure 1B), we have previously shown that blocking conjugation of SUMO or ubiquitin skews the NHEJ/HR balance in the opposite direction (22). Thus, we decided to analyze in more detail the role of protein neddylation in the choice between DSB repair pathways.

### Neddylation controls CtIP-mediated DNA end resection

Considering that cell cycle is a major control point for DSB repair, we discarded that the effect observed upon the use of the MLN4924 was due to change in cell cycle profile (Supplementary Figure S2). Only long treatments with MLN4924 changed cell cycle distribution. Thus, we restricted our experiments to short treatments with the inhibitor, unless otherwise specified.

We decided to test if protein neddylation has an impact on DNA end resection, the major control point between NHEJ and HR (5). We observed that the preincubation of cells with MLN4924 increased the number of cells with RPA foci induced by IR (Figure 1C). Thus, this hyper-resection phenotype suggested that protein neddylation acts as an inhibitor of DNA end resection. In agreement, overexpression of NEDD8 rendered the opposite results, i.e. reduced resection as measured by RPA foci (Figure 1D). As seen in Supplementary Figure S3A, a general increase of protein neddylation is observed in such conditions. Both, the increase in RPA foci formation and the increase in general protein neddylation are limited. Thus, although in agreement with our model NEDD8 overexpression skewed the balance toward a mild increase in NHEJ, it was not statistically significant (Supplementary Figure S4).

**Figure 2.**
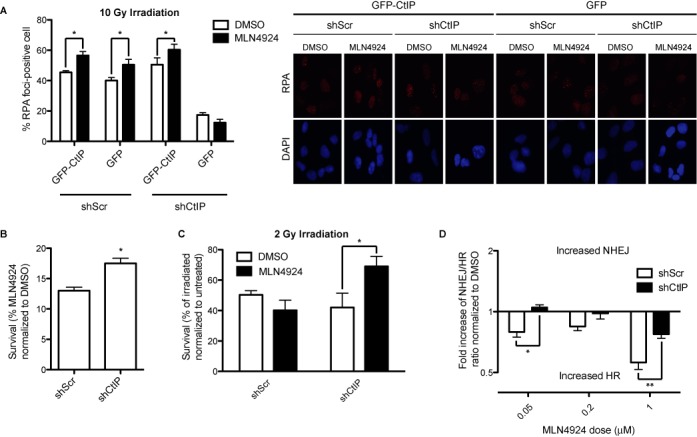
MLN4924 phenotypes in HR/NHEJ balance and DNA resection depend on CtIP. (**A**) Cells containing an shRNA against CtIP or a control shRNA (shScr) were transfected with an shRNA resistant GFP-CtIP fusion. One hour after MLN4924 or DMSO addition, cells were irradiated and an additional hour later the amount of cells showing RPA foci was scored. Other details are the same as in Figure 1C. (**B**) Cells expressing an shRNA against CtIP or control were treated for 12 days with MLN4924 0,1 μM or DMSO. The number of colonies formed in the presence of MLN4924 was normalized with the number of colonies in the DMSO control and plotted. Bars represent the average and standard deviation of three independent experiments. (**C**) Cells expressing the indicated shRNAs were irradiated with 2 Gy, with and without 1-h preincubation with 0.2 μM of MLN4924 or DMSO, then incubated 2 h with the inhibitor and then for 12 days in fresh medium. The number of colonies formed normalized with a control not irradiated is shown. Bars represent the average and standard deviation of three independent experiments. (**D**) The ratio between HR and NHEJ was calculated with the SSR system in cells expressing the indicated shRNAs and treated with different doses of MLN4924 as indicated. Details are the same as in (B). Statistical significance was calculated with a 2-way ANOVA.

Such an increase in resected DNA observed by neddylation inhibition was due to a hyperactivation of the canonical pathway, as it disappeared when cells were depleted of CtIP (Figure 2A; for an example of CtIP depletion see Supplementary Figure S3B). Indeed, depletion of endogenous CtIP without expressing any other form of CtIP (Figure 2A, cells transfected with GFP) showed a similar percentage of RPA-foci positive cells regardless of the addition of MLN4924 or the vehicle DMSO. Such effect was clearly dependent on CtIP as was complemented by the expression of a GFP-CtIP transgene, which cannot be targeted by the shRNA (Figure 2A). These data strengthen the idea that protein neddylation might inhibit HR by controlling CtIP-mediated resection.

To analyze the impact of the CtIP-mediated hyper-resection observed upon protein neddylation inhibition, we tested the viability of cells treated with MLN4924 in cells depleted of CtIP or control cells. As seen in Figure 2B, and in agreement with published reports (19), cells were sensitive to chronic treatments with MLN4924 compared with DMSO-treated cells in the absence of any exogenous DNA damage. However, if those cells were also depleted of CtIP, a mild but statistically significant increase in survival was observed (Figure 2B). Thus, neddylation inhibition toxicity was partially due to CtIP-dependent hyper-resection of endogenously aroused DNA breaks. Moreover, CtIP-depleted cells, but not control cells, were less sensitive to ionizing irradiation after a short treatment with MLN4924 (Figure 2C). Thus, our data suggested that the impairment on DNA resection caused by reduction of CtIP levels was partially corrected by the hyper-resection phenotype of neddylation inhibition (Figure 2C).

This observation that protein neddylation negatively regulates CtIP-mediated DNA end resection might explain why MLN4924 treatment skewed the choice between HR and NHEJ toward the former. To prove that the unbalance between HR and NHEJ observed upon neddylation inhibition was caused by excessive CtIP-mediated resection, we measured the balance between both repair pathways using the SSR in cells either mock treated with DMSO or treated with different doses of MLN4924 in combination with or without CtIP depletion. As seen in Figure 2D, shCtIP depletion prevents the hyper-recombination phenotype of MLN4924 addition, albeit only partially at higher doses.

### CtIP and BRCA1 interaction is controlled by neddylation

**Figure 3.**
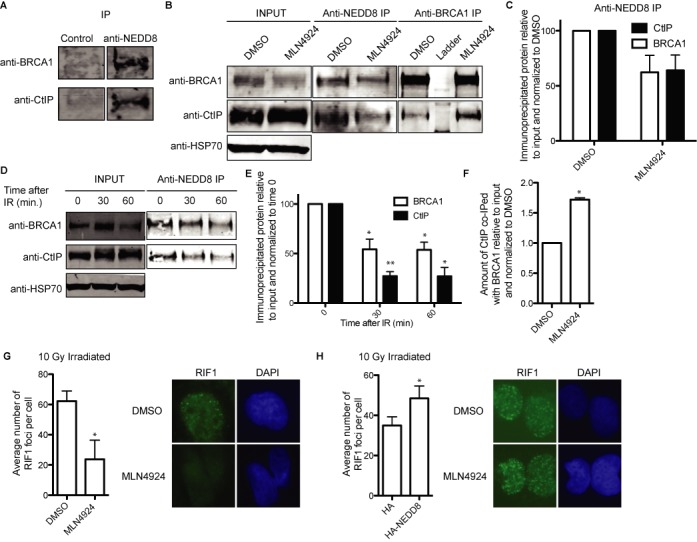
CtIP and BRCA1 complex formation is controlled by their interaction with neddylated proteins. (**A**) CtIP and BRCA interact with neddylated proteins. Protein extracts were divided into two and immunoprecipitated with an anti-NEDD8 antibody or a non-related IgG as a control and blotted with the indicated antibodies. A representative experiment is shown. (**B**) Protein samples from cells pre-treated with MLN2449 or DMSO were immunoprecipitated with either an anti-NEDD8 antibody or mix of two anti-BRCA1 antibodies and blotted with anti-CtIP or anti-BRCA1. A representative western blot is shown. (**C**) The amount of immunoprecipitated protein with anti-NEDD8 antibody from (B) was relativized to the input and then normalized to DMSO, taken as 100%. The average and standard deviation from three independent experiments is shown. (**D**) Cells were irradiated with 10 Gy and protein samples collected at the indicated time points. After immunoprecipitation with an anti-NEDD8 antibody, samples were blotted for CtIP and BRCA1. A representative western blot is shown. (**E**) Quantification of panel (D). The quantification of the amount of immunoprecipitated protein relative to the input and normalized to time 0 from three independent experiments is shown. (**F**) Quantification of BRCA1 IP from panel (B). Details are the same as in (C). (**G**) Average number of RIF1 foci per cells. Cells pre-treated with either DMSO or MLN4924 were immunostained with a RIF1 antibody. The graph represents the average and standard deviation of the number of foci per cell from three independent experiments. A representative image of each case is shown. (**H**) Same as (G), but cells transfected with HA-NEDD8 or an empty plasmid. The average of six independent experiments is shown.

In order to understand the molecular mechanism controlling the inhibition of DNA end resection through protein neddylation, we immunoprecipitated neddylated proteins using an anti-NEDD8 antibody. Then, we blotted for proteins that are important for DNA end resection and MLN4924 toxicity such as CtIP and BRCA1. Strikingly, we observed that both CtIP and BRCA1 were co-immunoprecipitated with an anti-NEDD8 antibody but not with a non-related control IgG (Figure 3A). However, when we blotted the same membranes with an anti-NEDD8 neither of those protein bands reacted with the antibody (data not shown). To be sure we were observing an interaction with neddylated proteins, we repeated the immunoprecipitation (IP) upon treatment with MLN4924, and we observed a reduction of the appearance of both CtIP and BRCA1 (Figure 3B; Anti-NEDD8 IP, and 3C). Thus, we conclude that neither CtIP nor BRCA1 is neddylated, or they are neddylated to such a low extent that we could not detect it. However, both of them separately or as a complex interact with neddylated proteins. Considering that protein neddylation is a potent inhibitor of DNA end resection, we decided to analyze the interaction of CtIP and BRCA1 with neddylated proteins after DNA damage. We observed that both CtIP and BRCA1 were readily immunoprecipitated with the NEDD8 antibody in untreated cells, but in both cases the amount immunoprecipitated was reduced after DNA damage (Figure 3D and E). CtIP and BRCA1 physically interact (27) and such interaction facilitates DNA end resection by eliminating RIF1 from the sites of DNA breaks (28,29). In fact, we have recently shown that BRCA1 interaction with CtIP controls the extent of resection at DSBs (26). Indeed, when we immunoprecipitated BRCA1, we observed that the amount of co-immunoprecipitated CtIP increased when protein neddylation was inhibited (Figure 3B and F). More importantly, when neddylation was hampered a sharp decrease in the number of RIF1 foci per cell after irradiation was clearly observed (Figure 3G). Supporting such results, overexpression of NEDD8 caused a mild, but statistically significant, increase of the average number of RIF1 foci per cell (Figure 3H). Thus, our results fit with the idea that some neddylated protein inhibits CtIP and BRCA1 interaction and, therefore, reduces DNA end-resection processivity by hampering the removal of RIF1 from the sites of the break. Moreover, such inhibition is eliminated by the appearance of DNA damage.

### The NEDD8 E2 UBE2M and E3 RNF111 block DNA end processing

**Figure 4.**
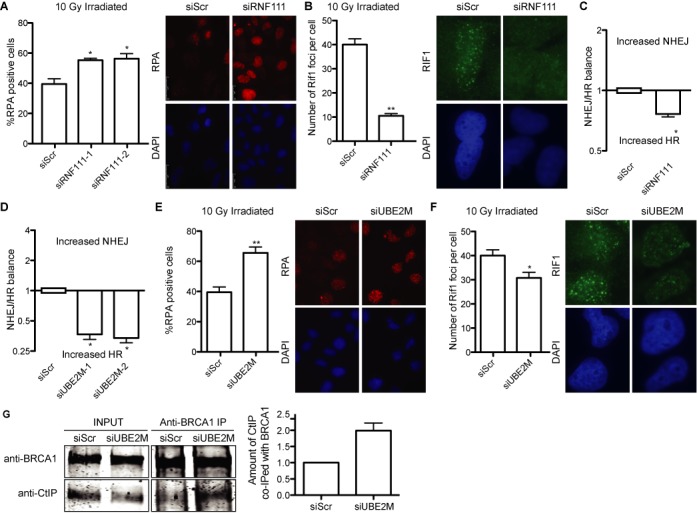
Role of RNF111/UBE2M in DNA end resection and DSB repair pathway choice. (**A**) Cells depleted for RNF111 or control cells were analyzed for RPA foci formation as described in Figure 1C. A representative immunofluorescence is shown. (**B**) Same as in (A), but cells were analyzed for RIF1 foci as described in the Materials and Methods section. (**C**) The balance between HR and NHEJ was calculated with the SSR system in cells transfected with siRNAs against the indicated genes or control siRNA (siScr). Other details are the same as in Figure 1B. (**D**) Same as in (C), but cells depleted for UBE2M. (**E**) Same as in (A), but cells depleted for UBE2M. (**F**) As in (B), but cells depleted for UBE2M. (**G**) Protein samples from cells previously downregulated for UBE2M were immunoprecipitated with a mix of two anti-BRCA1 antibodies and blotted with anti-CtIP or anti-BRCA1. A representative western blot and the quantification from five independent experiments, made as described in Figure 3, are shown.

Our data suggest that protein neddylation is a potent inhibitor of DNA end processing. Previously it has been shown that RNF111, an E3 ligase of the STUbL type, is involved in the DDR by facilitating neddylation and ubiquitylation of several targets (13,30). We analyzed the role of RNF111 in DNA end resection. As for neddylation inhibition with MLN4924 (Figures 1C and 3G), DNA resection was increased upon depletion of RNF111 as observed by an increase in RPA foci formation (Figure 4A; for an example of RNF111 depletion see Supplementary Figure S3C). The same results were observed with two different siRNA against RNF111 (Figure 4A). This was accompanied by a reduction in RIF1 foci (Figure 4B). As a consequence, and again in a similar way to MLN4924 addition, the balance between NHEJ and HR was skewed toward an increase in HR using the SSR reporter (Figure 4C). In order to exclude a role of RNF111-mediated ubiquitylation (30) in those phenotypes, we depleted UBE2M, the E2 enzyme that collaborates with RNF111 specifically in protein neddylation (13,20). In agreement with the role of RNF111 in DNA end resection and DSB repair pathway choice being dependent on its neddylation role, we observed an unbalance of DSB repair pathways toward HR (Figure 4D; see Supplementary Figure S3D for depletion of UBE2M). Similar results were observed with two different siRNA targeted against UBE2M. Such unbalance was caused by an increase in RPA foci and a reduction in RIF1 foci (Figure 4E and F). Thus, our results agree with an inhibitory function of RNF111/UBE2M-dependent neddylation at the sites of breaks over CtIP-mediated resection and HR. Indeed, co-immunoprecipitation of CtIP with BRCA1 was increased in the absence of UBE2M (Figure 4G), suggesting that such a role occurs, at least partially, at the level of BRCA1–CtIP interaction.

### Protein neddylation regulates DSB repair pathway choice by affecting the length of resected DNA

**Figure 5.**
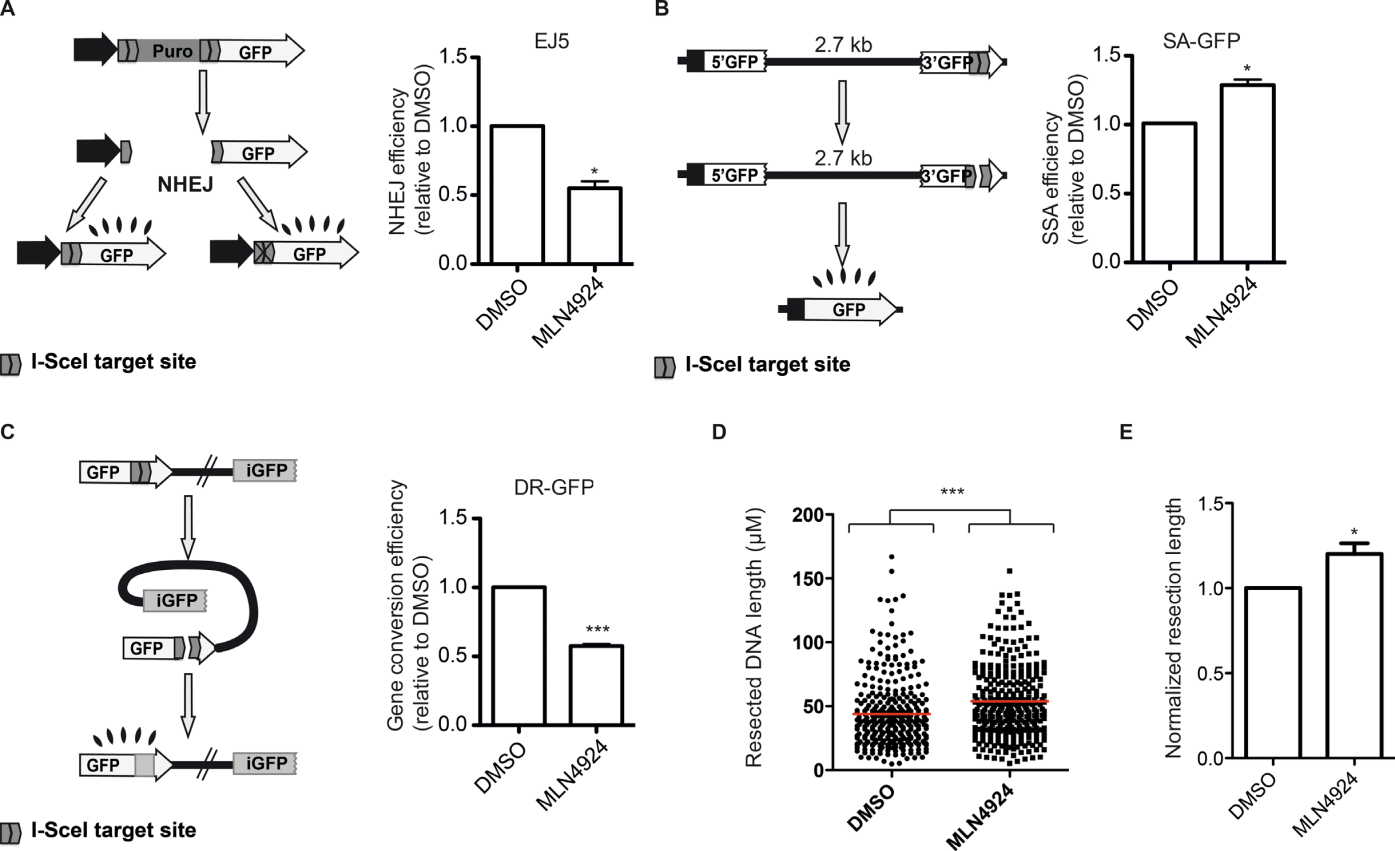
Effect of inhibition of protein neddylation in different DSB repair pathways. (**A**) In the EJ5 reporter (left), I-SceI-induced DSB can be repaired by NHEJ recreating an active GFP gene, containing or not a functional I-SceI target site. The percentage of green cells was calculated as described in the Materials and Methods section in cells pretreated with MLN4924 or DMSO. This percentage was normalized with the DMSO-treated cells value and plotted. Bars represent the average and standard deviation of three independent experiments. (**B**) Same as (A), but using the SA-GFP. Such a reporter is formed by two truncated copies of the GFP that, upon SSA, can restore an active GFP gene with the deletion of one of the repeats and the intervening region. Other details are the same as in (A). (**C**) The DR-GFP reporter is formed by two non-functional copies of the GFP. Gene conversion induced by an I-SceI-mediated DSB restores an active GFP gene. The efficiency of gene conversion was calculated as described in (A) for NHEJ. (**D**) Single molecule analysis of resection tracks (SMART) of cells treated with DMSO or MLN4924. The length of individual fibers is shown as a scatter plot. A Mann–Whitney test was performed to analyze the statistical difference of both populations. (**E**) The median length of resected DNA was normalized to DMSO. The average of four independent experiments is shown.

**Figure 6.**
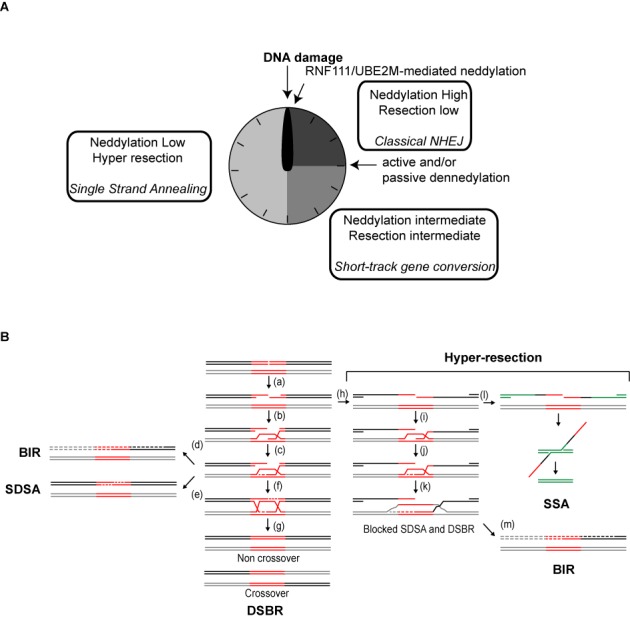
The extent of resection influences the outcome of recombination. (**A**) Neddylation acts as a molecular timer for DNA end resection and repair pathway choice. First, high local protein neddylation due to RNF111 activity inhibits resection favoring NHEJ. After that, NEDP1 and CSN activation reduce protein neddylation, allowing some resection to take place. Most breaks will be repaired by short-track gene conversion. Finally, local protein neddylation is sufficiently reduced to allow hyper-resection, favoring repair such as SSA that requires long tracks of resected DNA. (**B**) A broken DNA that is going to be engaged in recombination is resected (a) before the 3′ OH overhand is used to invade (b) a homologous region (red lines) located elsewhere (gray lines). DNA synthesis (dashed lines) will use the homologous DNA as a template (c) and it could continue until the end of the chromosome (d; BIR), the newly synthesized DNA can reanneal (e; SDSA recombination subpathway) or it can be ligated with the resected DNA to form two Holliday Junctions (f; DSBR recombination subpathway). Depending on how such structures are resolved, recombination will lead or not to crossing overs (g). However, hyper-resection (right) could lead to the exposure of ssDNA regions that are no longer homolog (h; black versus gray lines). In such scenario, although DNA invasion (i) and DNA synthesis (j–k) might happen, a non-homolog DNA would be used as a template for DNA synthesis (k; gray dashed lines). Thus, the newly synthesized DNA can no longer reanneal with the acceptor DNA (black solid line), effectively blocking SDSA and HJ formation. If additional repeated sequences are located nearby (green lines), the break can be sealed using additional recombination pathways such as SSA (l). An alternative would be that DNA synthesis continues until the end of the chromosome (m; BIR).

Our data suggest that protein neddylation has a major role controlling DNA end resection, hence controlling the appearance of ssDNA. Thus, it might regulate DSB repair pathway choice. To analyze this idea, we tested different DSB repair pathways upon protein neddylation inhibition with MLN4924. First, we used the previously published EJ5 reporter (24) to analyze NHEJ. In this reporter, an active GFP gene is formed when cells repair an I-SceI-induced DSB. As expected, NHEJ was inhibited due to an increase in DNA end resection (Figure 5A). The hyper-resection we observed by an increase on RPA foci can be due to two, not mutually exclusive, scenarios: an increase in the number of breaks that are resected (for example due to resection taken place in G1) and/or an increase in the length of resected DNA. Whereas in the first case all homology-driven DNA-repair mechanisms will be favored, in the second case hyper-resection will stimulate those pathways that require longer resection, such SSA, but would block some HR subpathways if resection reaches outside the homologous stretch of DNA (see the Discussion section for details; Figure 6B). Thus, we tested different HR pathways. As the SSR system used to initiate this study compares NHEJ with SSA, we first used the SA-GFP reporter that measures SSA (24). As expected, we observed that neddylation inhibition increases this particular repair pathway (Figure 5B). This increase in SSA and reduction in NHEJ explain the unbalance observed with MLN4924 addition with the SSR. Strikingly, not all HR subpathways were affected equally by neddylation inhibition. Indeed, gene conversion was diminished when the DR-GFP reporter (23) was used (Figure 5C). Hence, we hypothesized that neddylation is controlling the extent of resection and not the amount of breaks that are resected, favoring SSA over other HR subpathways. To prove it, we performed a SMART assay, that measures the length of resected DNA at the level of individual fibers (26), with cells treated with MLN4924 or DMSO. Although resection is an asynchronous process in the population, thus the length of resected DNA is heterogeneous, we observed that treatment with MLN4924 statistically changes the shape of the population (Figure 5D), and resected DNA tracks tend to be longer. In fact, we observed that neddylation inhibition causes a 20% increase in the median length of resected DNA (Figure 5E). Thus we conclude that protein neddylation controls the extent of resection and impacts in the repair mechanism that will be used to repair the break, but not only at the decision between NHEJ and HR but also at the choice between different homology-mediated repair pathways.

## DISCUSSION

Here, we have tested several small molecule inhibitors used in clinical trials and related with the DDR for their impact in the choice between NHEJ and HR. We observed that protein deacetylation has a great impact in repair pathway choice (Figure 1B). TSA skewed the balance toward HR. Affecting NHEJ, HR or both, something we could not discriminate with this genetic tool, could cause this unbalance. TSA inhibits specifically class I and II HDACs, such as HDAC1 and HDAC2, both of them previously related with DNA repair (31). Moreover, TSA has been previously shown to alter chromatin structure in a way that facilitates ATR activation (32). Thus, we propose that TSA effect on DSB repair pathway choice might be due to this altered chromatin structure that facilitates DNA end resection, hence increasing SSA and reducing NHEJ.

Another small molecule that alters the relative ratio between HR and NHEJ was the replication inhibitor aphidicolin (Figure 1B). Aphidicolin inhibits the DNA polymerase and, as a consequence, cells accumulate in S-phase. In agreement with HR being restricted to S and G2 phases of the cell cycle (5), such accumulation in S-phase renders a net increase of HR over NHEJ, as shown in Figure 1B.

We also tested an inhibitor of PARP due to its established relationship with DSB repair. PARP1 inhibition blocks single strand break repair. Those unrepaired breaks are carried out to the next S-phase where they are converted to DSBs, and HR has been proved essential to repair them (33). Moreover, PARP1 and PARP2 have been directly involved in NHEJ and HR choice (2). In addition, the newest member of the PARP family, PARP3, plays a role in DNA repair by HR and it has been shown to control DNA end resection (34). However, when we used a dose of PARP inhibitor that reduces the activity of all three PARP proteins, we did not see any effect in the SSR system. This might be due to a complex crosstalk between positive and negative effect of different PARP proteins in repair pathway choice.

Previously, others and we have shown that MRE11 is a major regulator of DSB repair pathway, probably due to its role in DNA end resection (2,5,22,35). MRE11 has different catalytic activities, such as 3′-5′ and 5′-3′ exonuclease and endonuclease. We used Mirin, a known inhibitor of the exonucleolytic activity of MRE11 (36). As there is no effect of Mirin on the SSR, we conclude that the exonuclease activity of Mre11 is not required for DNA end processing, in agreement with other reports that propose that resection by the MRN complex is based on the release of short ssDNA oligos produced by Mre11 endonuclease activity (35,37).

Ubiquitylation and sumoylation of proteins have a major role in the DDR and DSB repair (4) and we have shown that they mainly facilitate HR to take place (22). So, we tested the effect of another ubiquitin-like protein modifier, NEDD8, in DSB pathway choice. For that we used a neddylation inhibitor MLN4924 (38) and observed that exerted an effect similar to TSA (Figure 1B). Namely, neddylation inhibition, and contrary to blocking conjugation of Ubiquitin and SUMO (22), skewed the balance toward homology-mediated repair. Indeed, protein neddylation seems to be a potent inhibitor of DNA-end resection and HR. In agreement with our results, impairment of neddylation by MLN4924 treatment or depletion of the NEDD8 E2 ligase UBE2M has been shown to increase RAD51 foci (20). There have been some reports involving protein neddylation in the DDR (13,17,19–20). All of them showed a role of protein neddylation at the sites of breaks (13,17). Different histones (H2A, H4) and DDR-related Ubiquitin ligases (RNF8, RNF168) seem to be neddylated and such neddylation is required for proper DDR activation. Moreover, neddylation of specific cullins also impacts on the response to DNA damage (20). Our data suggest that one or several unknown proteins neddylated in a UBE2M-dependent manner limit the length of resected DNA by blocking the interaction between CtIP and BRCA1 (Figures 3 and 4G), effectively hampering RIF1 removal from damaged chromatin (Figure 3). This hypothesis agrees with the idea that CtIP–BRCA1 interaction modulates the extent and rate of DNA-end resection (26). We have not been able to observe neddylation of either CtIP or BRCA1, thus we propose that some unknown factor is affecting their interaction when is conjugated to NEDD8. One likely candidate is RNF168 that is known to be neddylated (17); its recruitment to damaged DNA is neddylation dependent (13) and controls BRCA1 retention at sites of DSBs (39). However, we cannot discard the contribution of other neddylated factors, including Cullins. Indeed, a relationship between CUL7 and CtIP has been recently shown in terms of pathology, as specific mutations in either gene are associated with primordial dwarfism with similar phenotypes (40,41). Although our data support that neddylation inhibits CtIP-mediated resection, we cannot discard that some of the observed phenotypes depend also on putative roles of neddylation in other steps of HR, such as the BLM/EXO1-dependent long-range resection, the strand annealing reaction, etc.

Previously reported neddylation at the sites of damaged DNA is dependent on the activity of RNF111 (13,17). RNF111 activity increases upon DNA damage and acts locally in the vicinity of the breaks. RNF111 and NEDD8 are recruited fast to the sites of breaks, with a peak at 10 min after DNA damage induction (13). After that, its accumulation fades slowly with time. Thus, local protein neddylation is indeed a quick response that fits with a role with a fast DNA repair such as NHEJ. Although we cannot exclude a role of the ubiquitylation function of RNF111 in the phenotypes observed, the fact that similar effects are observed with UBE2M depletion leads us to propose that the neddylation activity of RNF111/UBE2M is involved in DNA resection, hence HR, locally (Figure 4). Qualitatively RNF111 and UBE2M depletion showed similar phenotypes (Figure 4), but the differences in magnitude might suggest that both RNF111 and UBE2M have additional and independent roles controlling DNA end resection, something we cannot discard. In agreement with the general neddylation status controlling DNA end processing, overexpression of NEDD8 by itself reduces DNA end resection (Figure 1D). So, we propose that protein neddylation might regulate DSB repair acting as a molecular timer (Figure 6A). Early after DSB appearance, RNF111 accumulation would hamper DNA end resection favoring NHEJ. Later, neddylated proteins will lose NEDD8 due to passive and active mechanisms. On the one hand, RNF111 will be excluded from damaged chromatin (13). On the other hand, the action of the CSN complex and NEDP1 might actively contribute to protein deneddylation. In agreement with this model, it has been shown that both deneddylase activities are activated as a response to DNA damage. First, two subunits of the main deneddylase activity, the COP9 signalosome (CSN), are known to be an ATM substrate (21). Also, RNF168 is first neddylated after DNA damage, but this neddylation is transient and starts to drop after 30 min due to the action of the dennedylase NEDP1 (17).

This neddylation-dependent molecular timer will regulate the type of DSB repair that will take place by controlling the extent of DNA end resection (Figures 5D and 6). This agrees with neddylation modulating CtIP–BRCA1 interaction that we have shown controls the length of resected DNA (26). First, the accumulation of RNF111-mediated neddylation will favor classical NHEJ. Some of those breaks will not be suitable for NHEJ, probably due to modification of the DNA ends and will remain unrepaired longer. As soon as protein neddylation starts to disappear, probably by both RNF111 eviction and CSN and NEDP1 activation, some resection of the break will take place. This limited processing will ‘clean’ the DNA ends and might activate short track MMEJ. Even then some breaks might stay unrepaired. Further protein deneddylation of proteins will facilitate CtIP and BRCA1 interaction, increasing resection processivity by facilitating RIF1 removal and the length of resected DNA. The exposed ssDNA will be engaged in HR.

During recombination with an ectopic sequence, as in the recombination measured by the DR-GFP reporter, it is essential that resection is limited within the homolog region (Figure 6B). Only then, non-mutagenic subtypes of recombination such as DSBR and SDSA can take place (Figure 6B, a–g). However, in the complete absence of protein neddylation, as with MLN4924 treatment, hyper-resection will occur, i.e. resected DNA will be longer (Figure 6B, h). Then, both DSBR and SDSA are effectively blocked (Figure 6B, i–k). This explains our observation that short track gene conversion with the DR-GFP reporter is reduced upon inhibition of protein neddylation (Figure 5C). In agreement, RNF111 and UBE2M depletion have also been shown to reduce gene conversion on the DR-GFP reporter despite the later increasing RAD51 foci formation (13,20). Thus, hyper-resection caused by lack of protein neddylation will reduce not only NHEJ but also short-track gene conversion, and will switch the repair profile toward pathways that are favored by long resected tracks such as SSA (Figure 6B, l). Additionally, BIR might happen independently of the extent of resected DNA (Figure 6B, d and m). In any case, hyper-resection favors mutagenic recombination pathways and might potentially lead to deletions or loss of heterozygosis.

In principle, this limitation in the extent of resected DNA required for gene conversion will not apply to recombination with the sister chromatid, as the donor and acceptor molecules are completely identical. However, even in this case hyper-resection will reduce the chances of the newly synthesized DNA to catch up with the resected 5′ end to form an HJ (42). Moreover, it will increase the probability of uncovering repeated sequences that can be the substrate for an SSA-type of recombination. Thus, complete deneddylation, such as the one obtained with MLN4924 addition, albeit not completely blocking gene-conversion when the sister chromatid is used as a template, will facilitate the mutagenic SSA subpathway even in this condition. Hence, we propose that protein neddylation controls DSB repair pathway choice, the decision not only between NHEJ and HR but also between different recombination subpathways. Accordingly with previous results and our observations, we propose that a wave of protein neddylation and deneddylation controls the timing of DNA end resection, thus establishing a temporal organization between different repair types.

This relationship between protein neddylation and DSB repair can explain the potent anticancer activity showed by MLN4924 in pre-clinical studies (38). Moreover, considering that HR is defective in many human tumors, it is worth to speculate that MLN4924 will be especially successful in treating those cancers. In them, MLN4924 will channel endogenous DNA breaks toward the impaired HR repair. Thus, we speculate that HR-deficient tumors might be hypersensitive to MLN4924. However, this will apply only to those cancer cells defective in HR steps that take place after DNA end resection. On the contrary, our data suggest that cells impaired in DNA end resection, such as depleted for CtIP, are resistant to MLN4924 treatment (Figure 2B). Interestingly, a reduction of MLN4924 toxicity has also been observed when other proteins involved in DNA end resection such as BLM or BRCA1 are depleted (43,44). In addition, our data suggest that in resection-deficient backgrounds MLN4924 protects cells from irradiation (Figure 2C). Thus, we postulate that MLN4924 treatment in tumors with reduced amounts of resection proteins such as CtIP and BRCA1 will reduce the efficiency of radiotherapy. As both proteins are known to be downregulated in certain tumors (45,46), it will be important to know the genetic contribution of different recombination genes to specific tumors to evaluate the potential effect of MLN4924 as an anticancer drug.

## SUPPLEMENTARY DATA

Supplementary Data are available at NAR Online.

SUPPLEMENTARY DATA
