# Supplementary material for: Neddylation inhibits CtIP-mediated resection and regulates DNA double strand break repair pathway choice
Source: Nucleic Acids Res. 2015 Jan 7;43(2):987–99. doi: 10.1093/nar/gku1384 (PMC4333419; doi:10.1093/nar/gku1384)
Supplement: SUPPLEMENTARY DATA [file supp_43_2_987__index.html]

Neddylation inhibits CtIP-mediated resection and regulates DNA double strand break repair pathway choice — SUPPLEMENTARY DATA 

# Neddylation inhibits CtIP-mediated resection and regulates DNA double strand break repair pathway choice

## SUPPLEMENTARY DATA

**Files in this Data Supplement:**

- SUPPLEMENTARY DATA
