# Supplementary material for: Neddylation inhibits CtIP-mediated resection and regulates DNA double strand break repair pathway choice
Source: Nucleic Acids Res. 2015 Jan 7;43(2):987–99. doi: 10.1093/nar/gku1384 (PMC4333419; doi:10.1093/nar/gku1384)
Supplement: SUPPLEMENTARY DATA [file supp_gku1384_nar-03199-d-2014-File002.pdf]

Supplementary Methods:

Cell cycle analysis

Cells were plated and incubated at 37ºC in complete DMEM medium. 24 hours later, they were treated with 0,2 μM MLN4924 for 1, 2 or 24 hrs, as indicated. After collecting the cells with trypsin, they were resuspended in 1 ml PBS and washed twice in 1 ml PBS. Then, cells were fixed in 5 ml 70% ethanol and incubated at 4ºC for 2 hrs, washed twice with PBS, resuspended in 0,2 μl of 1 mg/ml of propidium iodide and RNAse 0,2 mg/ml and incubated a 37ºC for 30 minutes. At least 10000 cells were counted from each sample.

Supplementary legends

Figure S1: An example of cells positive (solid arrows) or negative (empty arrows) for RPA foci.

Figure S2: Cell cycle distribution of U2OS cells treated with DMSO or MLN4924 for the indicated length of time. Cell cycle distribution was determined by FACS.

Figure S3: Protein depletions. A, Protein extracts were blotted with an anti-NEDD8 antibody to analyze the overall neddylation status in the cells. The signal of the whole lane, normalized to the loading control and relative to the transfection with the empty plasmid was calculated. The average and standard deviation of three independent experiments is shown. B, CtIP levels upon shRNA-mediated downregulation of CtIP. C, siRNA-dependent depletion of RNF111 using two alternative siRNAs compared with control. D, same as C, but depletion of UBE2M. An asterisk marks an unspecific band.

Figure S4: Effect of NEDD8 overexpression on the NHEJ/HR balance. Cells harboring the SSR system were transfected with HA-NEDD8 or an empty HA vector. The NHEJ/HR ratio was calculated as described and normalized to the cells with the empty vector.

Supplementary tables

Table S1: Inhibitors and concentrations used in the SSR screening

Inhibitor	Concentration	Supplier	Reference
**Thrichostatin A (TSA)**	1.3 µM	Sigma	T8552
**Aphidicolin**	1 µM	Sigma	A0781
**Mirin**	25 µM	Tocris	3190
**PARPi**	1 µM	AstraZeneca	Olaparib
**MNL4924**	1 µM	Vitro	I-502-01M

Table S2: siRNAs used in this study

Target gene	Description	Supplier	Reference
**Control**	ON-Targetplus Non-targeting pool	Dharmacon	D-001810-10-20
**RNF111-1**	GCGCUUCCAUUAACAAUUC	Dharmacon	J-007002-07-0010
**RNF111-2**	GAGUUGAGAUGAUUAAUAG	Dharmacon	J-007002-08-0010
**hUBE2M-1**	CAGAGGUCCUGCAGAACAA	Sigma	**(7)**
**hUBE2M-2**	GGGCUUCUACAAGAGUGGGAAGUUU	Sigma	**(14)**

Table S3: shRNAs used in this study

Target gene	Description	Supplier	Reference
**Scramble**	pLKO.1-puro Non-Target shRNA Control Plasmid DNA	Sigma	SHC016
**CtIP**	TRCN0000005403	Sigma	NM_0022894.1-3008s1c1

Table S4: PrimaryAntibodies used in this study. IF, immunofluorescence. WB Western blotting. IP, Immunoprecipitation. SMART, Single Molecule Analysis of Resection Tracks

Target protein	Application	Supplier	Reference
**NEDD8**	IP	GeneTex	GTX61205
**RNF111**	WB	Abnova	H00054778-M05
**BRCA1**	IP	Santa Cruz	sc-642
**BRCA1**	IP, WB	Santa Cruz	sc-6954
**HSP70**	WB	Santa Cruz	sc-24
**Tubulin**	WB	Sigma	T9026
**RPA32**	IF	Abcam	ab2175
**RIF1**	IF	Bethyl Laboratories	A300-568A
**Non related Rabbit IgG**	IP	Sigma	I8140

**Non related Mouse IgG**	IP	Sigma	I8765
**CtIP**	WB	R.Baer	
**UBE2M**	WB	Bostom Biochem	A-655
**BrdU**	SMART	Sigma	B5002-100MG

Table S5: Secondary antibodies used in this study. IF, immunofluorescence. WB Western blotting.

Antibody	Source	Application
**Alexa Fluor 594 goat anti-mouse**	Invitrogen	IF
**Alexa Fluor 488 goat anti-rabbit**	Invitrogen	IF
**Alexa Fluor 647 goat anti-mouse IgG**	Invitrogen	IF
**Alexa Fluor 568 goat anti-rabbit**	Invitrogen	IF
**IR-Dye 680 RD goat anti-mouse IgG (H+L)**	Li-Cor	WB
**IR-Dye 800CW goat anti-rabbit IgG (H+L)**	Li-Cor	WB

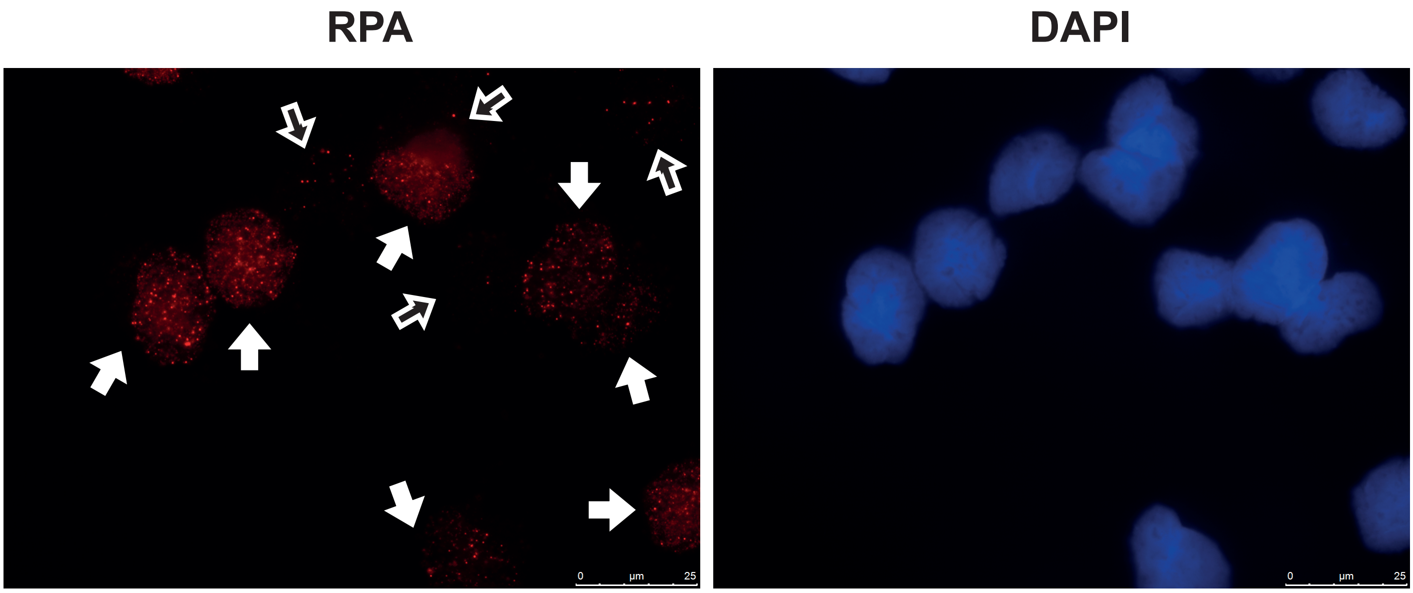

Jimeno *et.al.* Figure S1

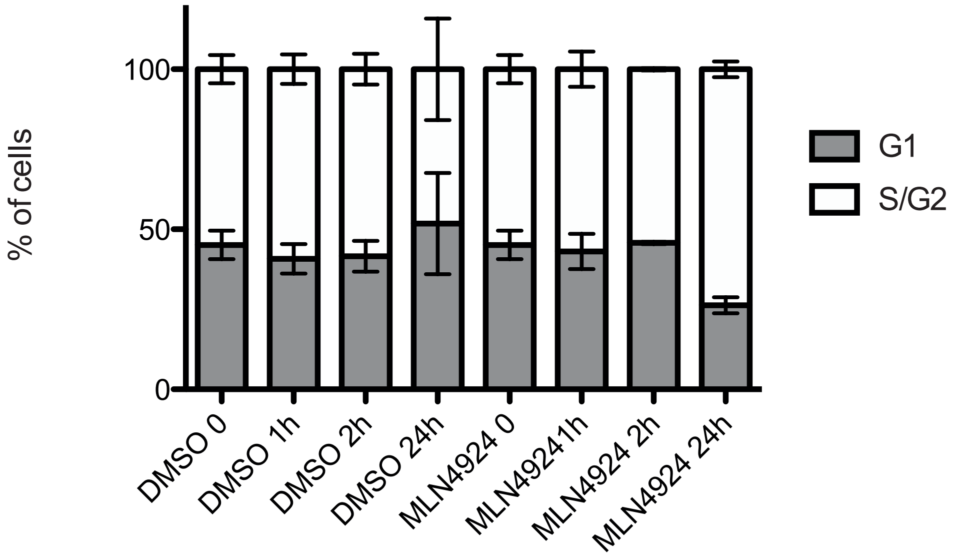

Jimeno *et.al.* Figure S2

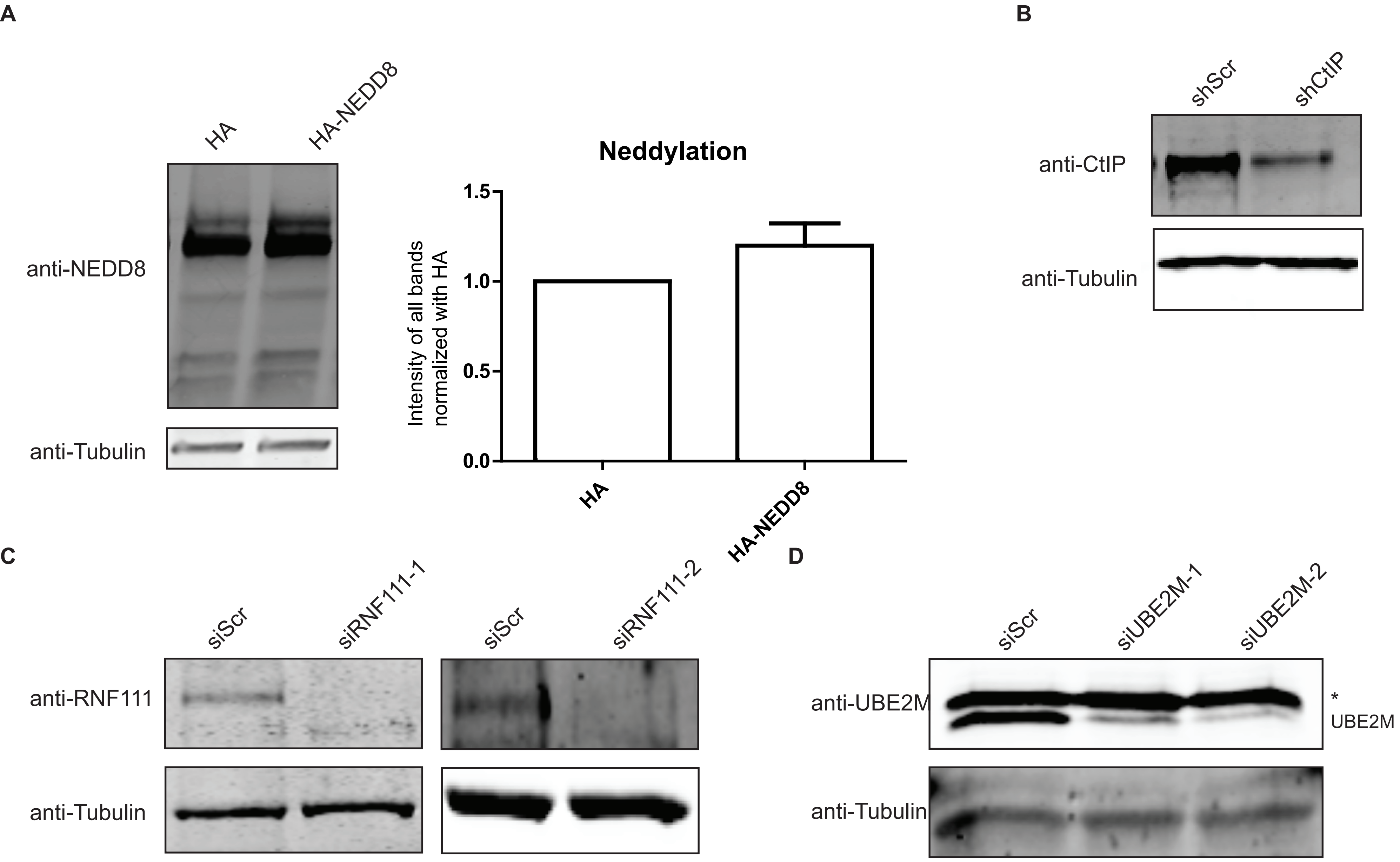

Jimeno et al Figure S3

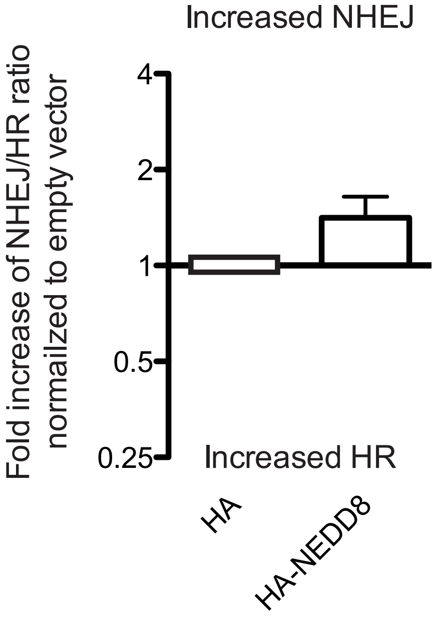

Jimeno *et.al.* Figure S4
